# Process Design for Optimized Respiration Identification Based on Heart Rate Variability for Efficient Respiratory Sinus Arrhythmia Biofeedback

**DOI:** 10.3390/ijerph19042087

**Published:** 2022-02-13

**Authors:** Jung-Nyun Lee, Min-Cheol Whang, Bong-Gu Kang

**Affiliations:** 1 Research Institute of Industrial Technology Convergence, Korea Institute of Industrial Technology (KITECH), Ansan 15588, Korea; blueleen2@kitech.re.kr; 2 Department of Human-Centered Artificial Intelligence, University of Sangmyung, Seoul 03016, Korea; whang@smu.ac.kr

**Keywords:** optimized respiration, heart stabilization indicator (HSI), heart rate variability (HRV), RSA biofeedback

## Abstract

Respiratory sinus arrhythmia (RSA) is a phenomenon in which the heart rate (HR) changes with respiration, increasing during inspiration and decreasing during expiration. RSA biofeedback training has an effect in relieving negative mental conditions, such as anxiety and stress. Respiration is an important indicator affecting the parasympathetic activation within the body during RSA biofeedback training. Although there are existing studies that consider individual differences when selecting optimized respiration using heart rate variability, the studies that use the high frequency components of HRV, which is an indicator of parasympathetic activation, are insufficient. For this reason, this paper proposes a process to identify optimized respiration for efficient RSA feedback, consisting of three steps: (1) application, (2) optimization, and (3) validation. In the application phase, we measured PPG data against various respiratory cycles based on the HF components of HRV and calculated the proposed heart stabilization indicator (HSI) from the data. Then, we determined the optimized respiration cycle based on the HSI in the optimization step. Finally, we analyzed seven stress-related indices against the optimized respiration cycle. The experimental results show that HSI is associated with the parasympathetic nervous system activation, and the proposed method could help to determine the optimal respiratory cycle for each individual. Lastly, we expect that the proposed design could be used as an alternative to improving the efficiency of RSA biofeedback training.

## 1. Introduction

Occupations that require much psychological attention over a long time (e.g., telemarketers and service personnel) can cause emotional and psychological distress. Activities [1,2,3] such as meditation (e.g., yogic therapy) can help to reduce the level of this stress in individuals. Specifically, yoga therapy uses spontaneous breathing and optimal breathing to maintain homeostasis of relaxation and rest. Studies on yoga breathing have shown that the practice of deep breathing (DB) strongly affects the human nervous system, especially the parasympathetic nervous system [2,4,5]. When people naturally breathe normally, they breathe relatively shallowly compared to deep breaths, and previous studies have shown that deep breathing has physiological effects over shallow breathing [6,7,8]. We call the fluctuations of the R–R interval RSA during this respiratory cycle, which means a cardiopulmonary response that changes the heart rate. The RSA is related to parasympathetic nervous activity, which is regulated by the vagal tone that changes heart rate variability (HRV) through activation [9,10,11,12,13,14].

Biofeedback training enables individuals to maintain and restore autonomic nervous system balance by regulating the activation of their physiological activities [15,16]. Bio-signals, such as the electroencephalogram (EEG), electrocardiogram (ECG), respiration (RSP), and blood volume pulse (BVP), can measure these physiological activities’ activation [17,18,19,20,21,22]. Specifically, biomarkers for utilizing feedback criteria are extracted, such as the frequency band of brainwaves, the heart rate respiratory cycle, and the HRV, by bio-signal acquisition and signal processing according to each signal feature. In addition, biofeedback training is particularly useful not only for managing stress, but also for managing a variety of forms of psychological distress that can be exacerbated through stress, such as anxiety, depression, attention deficit, and mental health conditions [23,24]. Additionally, it helps to provide opportunities for a continuous and natural transition from a stressful to a comfortable condition in one’s daily life by enabling people to control their own responses in stressful situations, and therefore, it is widely used in clinical treatment and the healthcare field. Some studies showed that biofeedback and neurofeedback have a medical effect on depression, anxiety disorders, and PTSD in adults [25], and have a clinical effect on ADHD and schizophrenia in psychiatric rehabilitation [26].

In particular, RSA biofeedback training, one of the types of biofeedback training, allows the body to become more stable by increasing parasympathetic activity. It leads people to breathe at the rate of the heart’s rhythm and baroreflex activity is associated with breathing [11,27,28]. The baroreflex is a reflex mediated by blood pressure sensors in the aorta and the carotid artery to modulate blood pressure fluctuations [29]. Baroreceptors in the arterial wall sense that the arteries are stretched, and when blood pressure increases, the baroreflex immediately decreases the heart rate. Likewise, the baroreflex has dynamic characteristics, and the sensitivity of the baroreflex can be described by the heart rate response to changes in blood pressure, i.e., HRV [30].

Researchers suggest that high HRV power promotes autonomic homeostasis and the ability to regulate emotionally [31], whereas low HRV power is associated with post-traumatic stress disorder (PTSD), depression [32], and insomnia [33]. Some studies to reduce the effects of these disorders show that HRV (RSA) biofeedback training can reduce symptoms of depression [34]. For this reason, HRV has been mainly studied in psychophysiology studies. Previous studies observed the change between successive R intervals, which implies a “dynamic map” of the correlation between the parasympathetic and sympathetic nerves of the autonomic nervous system [35,36].

Most studies have observed that a training rhythm of about six breaths per minute increases the power of the HRV [21,37,38,39], and thereby it has been a starting point to achieve optimization in individuals. On the other hand, other studies have argued that there is a difference between individuals, ranging from about four to seven breaths per minute [38,39,40,41,42,43]. Although the HF components of HRV are an important factor when monitoring parasympathetic activation, studies on selecting optimal respiration based on HF components are insufficient.

Therefore, this study proposes an overall process consisting of three processes—(1) application, (2) optimization, and (3) validation—to find an optimized breathing cycle to enhance the RSA biofeedback effect. The first process includes the breathing guidelines, including a visualization program to extract the proposed heart stabilization indicator (HSI). The second process explains selecting the optimal breathing cycle for each individual based on the indicator. The last process validates the proposed optimization method using the stress-related frequency domain parameters of HRV obtained from the experimental results.

This study is organized as follows. Section 2 describes the overall process for the HF components of HRV-based optimal respiration determination. Section 3 explains the experimental design and results of the application and optimization process, and Section 4 verifies the effect of optimized respiration, corresponding to the validation process. Section 5 explains and discusses the experimental results. Finally, Section 6 presents the conclusion.

## 2. Proposed Work

### 2.1. Overall Process Description

**Figure 1:**
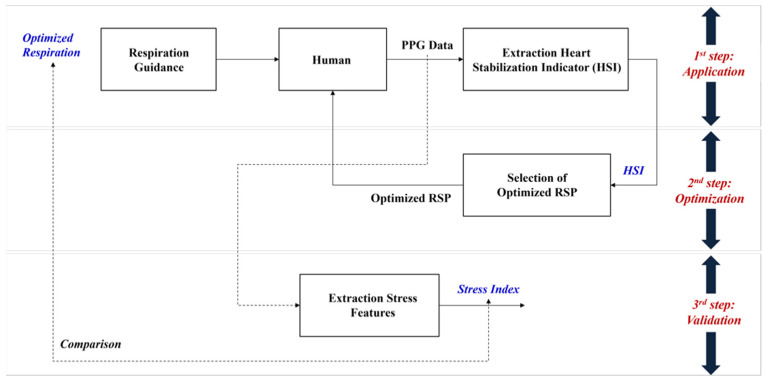
Overall process of proposed optimization on the respiration based on the HRV.

We proposed a process to find the optimized respiration cycle for an individual based on the HF components of HRV, consisting of three steps: (1) application, (2) optimization, and (3) validation, as shown in Figure 1. In the application step, we asked participants to breathe in five respiratory cycles (9 s, 10 s, 11 s, 12 s, and 13 s with half inhalation and half exhalation). For this, we provided a visual guideline for them to breathe for 2 min, and then allowed them to rest for the same amount of time they breathed. After a total of 5 respiratory cycles, we calculated the HSI, a suggested index, based on the PPG data measured during the respiratory period. In the optimization phase, we determined the optimal respiratory cycle for each individual through an analysis of HSI. Finally, we verified whether the optimized breathing cycle affects relaxation based on the seven stress-related features of the HRV frequency domain.

### 2.2. Description on Respiration Guidance

**Figure 2:**
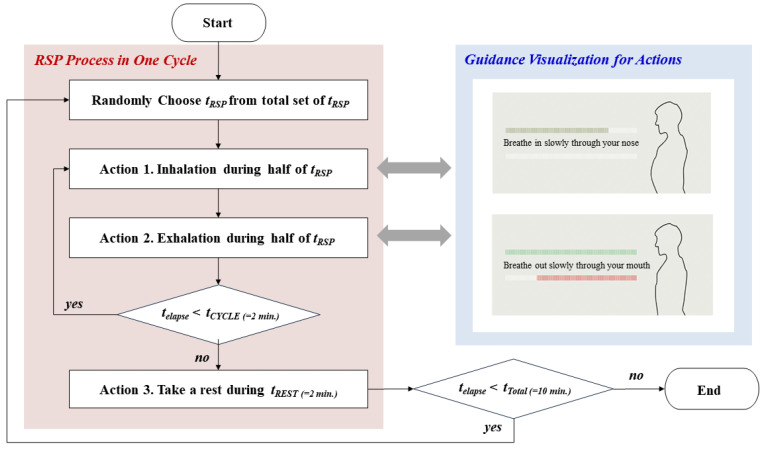
Detailed process on optimized respiration guidance including visualization.

In order to secure the reliability of the collected PPG data, this paper defines specific respiration guidelines, as shown in Figure 2. As shown in the red shaded area, we randomly selected each respiration cycle (*t_RSP_*) to eliminate the effect of the stimulation sequence on the experiment. We configured the same inhalation and exhalation time (half of *t_RSP_*) and gave the resting time (*t_REST_*) after a breathing cycle for 2 min (*t_CYCLE_*) as Actions 1 and 2. We also developed a visual screen as the blue part under the LabVIEW environment for apparent execution of these actions and showed it to the participants. The experiment lasted for 10 min (*t_Total_*) in a space free from environmental noise.

### 2.3. Description on Extraction HSI Process

**Figure 3:**
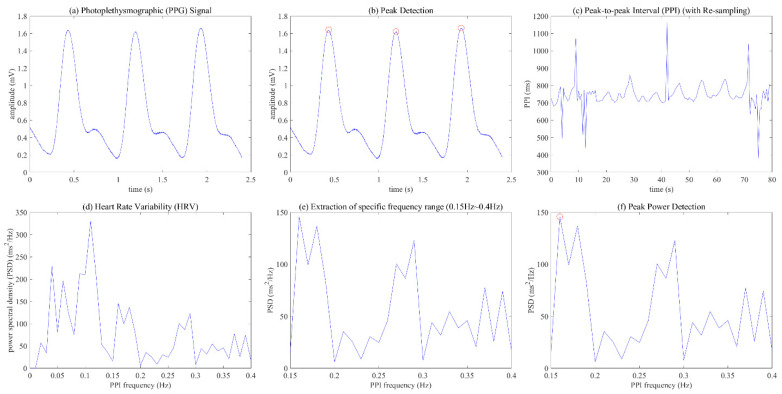
Signal processing process from the photoplethysmographic (PPG) data to the heart stabilization indicator (HSI): (**a**) PPG signal; (**b**) peak detection; (**c**) peak-to-peak interval (PPI); (**d**) heart rate variability (HRV); (**e**) extraction of specific frequency range (0.15 Hz~0.4 Hz); (**f**) peak power detection.

**Figure 4:**
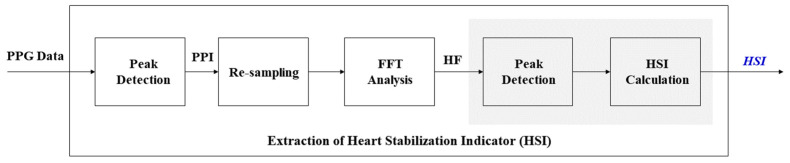
Detailed process on the extraction of the heart stabilization indicator (HSI) from PPG data.

To analyze meaningful results from PPG data, this paper defines the HSI indicator and HSI extraction process, and Figure 3 and Figure 4 show the detailed signal processing process to derive HSI from PPG. This process first detects the peak value in the PPG signal measured during respiration and calculates the peak-to-peak interval (PPI), as shown in (a) to (c) of Figure 3. Then, the process converts the 2 Hz resampled PPI into HRV frequency data using fast Fourier transform (FFT), and then selects the power in the range of 0.15~0.4 Hz as the high frequency (HF) power, as shown in (d) to (f) of Figure 3. Finally, we derive the HSI using the data in the HF components and Equation (1).

(1) $$\mathrm{HSI}=\frac{\mathrm{Peak}\;\mathrm{of}\;\mathrm{components}\;\mathrm{in}\;\mathrm{HF}\;\mathrm{range}-\mathrm{Mean}\;\mathrm{of}\;\mathrm{components}\;\mathrm{in}\;\mathrm{HF}\;\mathrm{range}\;}{\mathrm{Standard}\;\mathrm{Deviation}\;\mathrm{of}\;\mathrm{components}\;\mathrm{in}\;\mathrm{HF}\;\mathrm{range}}$$

### 2.4. Description on Extraction Stress Features

**Figure 5:**
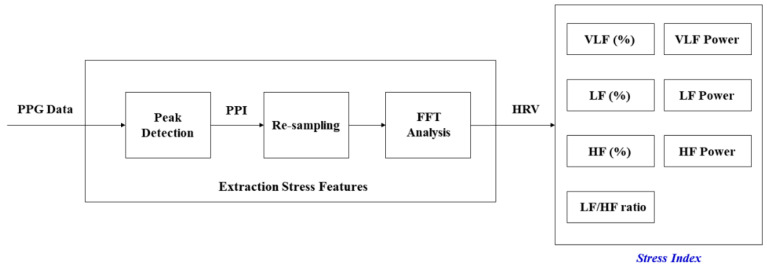
Detailed process on the extraction of the stress index from PPG data.

In order to verify the effect of the optimized breathing cycle on body stress, this paper defined seven stress-related indicators, and Figure 5 shows the process of extracting stress-related indicators from PPG data. Some of the processes in Figure 5 are similar to the unshaded parts in Figure 4. We recorded the PPG data at a sampling frequency of 500 Hz during the experiment and calculated seven features: VLF (%), LF (%), HF (%), VLF power, LF power, HF power, and LF/HF ratio.

## 3. Experimental Design

### 3.1. Participants and Experimental Device

We recruited 100 participants who worked in telemarketing (28 male, 72 female), aged between 20 and 50 years (mean = 34.09, SD = 7.59). The population ratio of participants was, respectively, 34%, 39%, and 27% for those in their 20s, 30s, and over the age of 40. The participants were physically healthy, and we gave them a small amount for active participation in the experiment. The average employment period was 11.8 months (SD = 14.7).

The subjects worked a total of 8 h a day, 40 h per week. Additionally, when examining the characteristics of the subjects through the interview, the subjects did not usually have time to perform activities for a long time, and there were a total of four subjects who practiced breathing training such as yoga and abdominal breathing. The other subjects had no personal breathing training experience, and all subjects performed three training sessions in this experiment. We used the PPG sensor with a BIOPAC amplifier (Model PPG 100C) to record the PPG. We transferred the amplified signal to a LabVIEW platform via a National Instrument DAQ-6015 AC/DC converter. The measured location of the sensor was on the index finger of the left arm.

### 3.2. Stimuli (Respiratory Cycles)

We asked the participants to breathe using the presented visual guide of respiratory cycles. The respiratory cycles consisted of five stimuli with 9 s, 10 s, 11 s, 12 s, and 13 s, where each respiratory cycle consisted of inhaling and exhaling. For example, they repeated breathing in and out for 6.5 s each for 2 min when we asked subjects to perform the 13 s breathing cycle.

### 3.3. Dependent Variables

We analyzed seven features, namely the percent and power of very low frequency (VLF), the percent and power of low frequency (LF), the percent and power of high frequency (HF), and the LF/HF ratio. The frequency band of VLF was 0.0033~0.04 Hz, the LF band was 0.04~0.15 Hz, and the HF band was 0.15~0.4 Hz.

### 3.4. Statistical Analysis

All dependent variables adhered to the within-subject design, and we performed non-parametric tests because they had non-normal distribution. The data analysis was performed based on the Wilcoxon signed-rank test between optimal RSP and none-optimal RSP.

## 4. Experimental Results

### 4.1. Relationship HSI and Stress Indices

**Figure 6:**
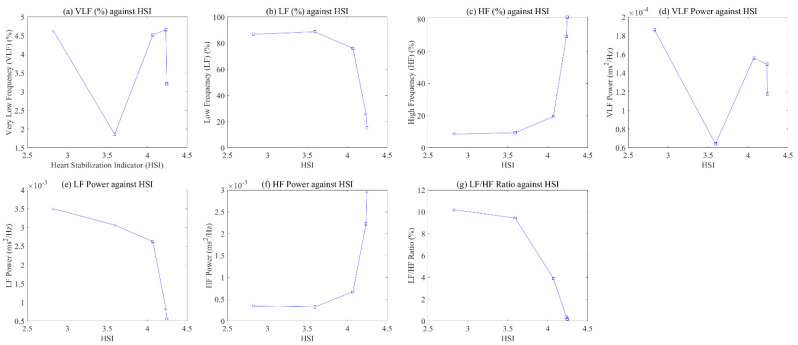
Relationship between HSI and 7 stress indices: (**a**) VLF; (**b**) LF; (**c**) HF; (**d**) VLF power; (**e**) LF power; (**f**) HF power; (**g**) LF/HF ratio.

Figure 6 shows the relationship between stress indices extracted based on HSI and HRV for one of the participants. The *x*-axis and *y*-axis represent the HSI and seven indicators, respectively. We confirmed the change in each indicator as the HSI value increased. Figure 6 shows a visually clear pattern in each indicator, except for VLF (%) and VLF power. As HSI increased, the LF (%), LF power, and LF/HF ratio indicating sympathetic nervous system activation decreased, while HF (%) and HF power indicating parasympathetic activation increased.

**Figure 7:**
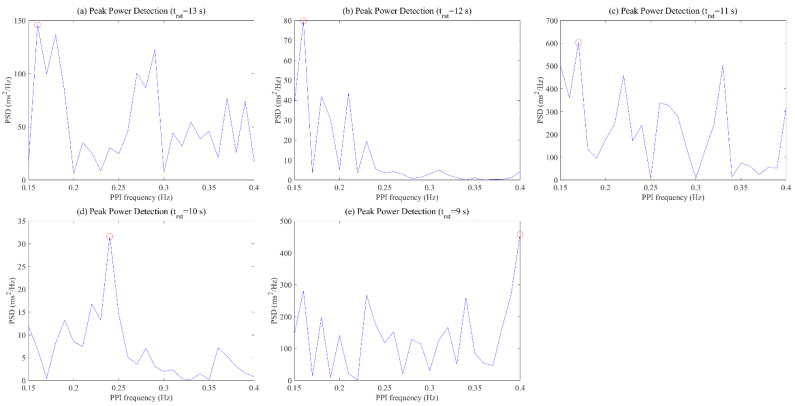
HF frequency pattern according to respiratory cycles: (**a**) 13 s RSP cycle; (**b**) 12 s RSP cycle; (**c**) 11 s RSP cycle; (**d**) 10 s RSP cycle; (**e**) 9 s RSP cycle.

Figure 7 shows the frequency pattern of the HF range calculated through the PPI data at this time. When the respiratory cycle was 13 s, 12 s, 11 s, 10 s, and 9 s, the HSI was 3.06, 4.07, 1.95, 4.24, and 1.95, respectively. The optimal respiration cycle of the participant was selected as the 10 s respiration cycle (=the highest HSI) and showed a dominant pattern at a specific frequency over other respiration cycles. On the other hand, other respiratory cycles resulted in multiple congested patterns rather than the dominant pattern at a specific frequency in the HF range.

### 4.2. Difference of Optimal and Non-Optimal RSP

**Figure 8:**
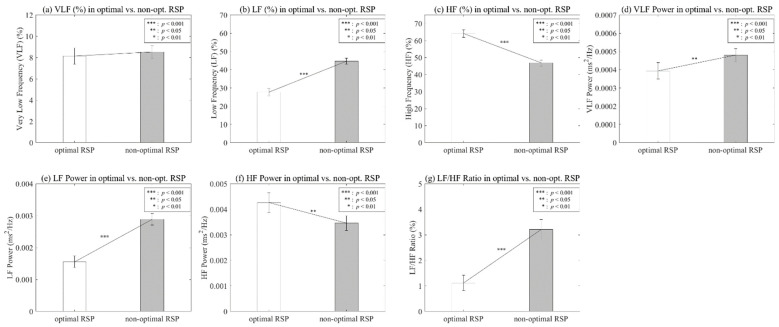
Analysis on HRV frequency parameters according to optimal vs. non-optimal RSP.

To verify the effect on optimal respiration, we analyzed the seven features of HRV frequency, and Table 1 and Figure 8 show the statistical results. We confirmed a statistical difference among these features, except for VLF (%).

The LF (%) showed a significant main effect of optimal RSP, *p* < 0.001, indicating a difference between optimal RSP (M = 27.7045, SD = 19.4434) and non-optimal RSP (M = 44.6741, SD = 16.2995). HF (%) also showed a significant result, *p* < 0.001, indicating a difference between optimal RSP (M = 64.1596, SD = 22.2799) and non-optimal RSP (M = 46.806, SD = 17.6863).

The VLF and HF power showed a statistically significant difference (*p* < 0.05). The mean and SD of the optimal respiration of VLF power were 0.0004 (SD = 0.0004), and otherwise 0.0005 (SD = 0.0003). Those of HF power were 0.0043 (SD = 0.0038) for optimal breathing and 0.0035 (SD = 0.0029) for non-optimal breathing.

The LF power and LF/HF ratio showed a significant difference (*p* < 0.001). The mean and SD of the optimal respiration of LF power were 0.0016 (SD = 0.0018), and otherwise 0.0029 (SD = 0.0018). Those of the LF/HF ratio were 1.1185 (SD = 3.021) for optimal breathing and 3.2043 (SD = 3.9283) for non-optimal breathing.

**Figure 9:**
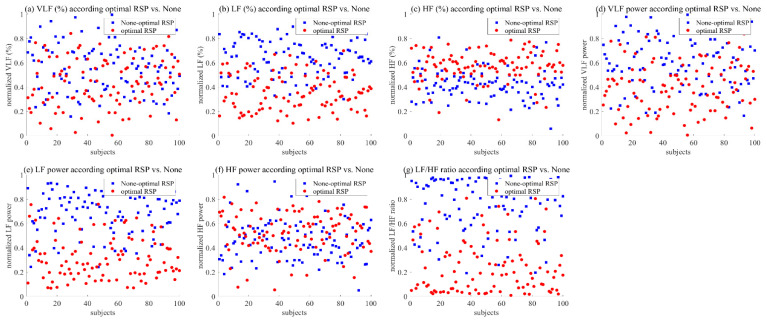
Pattern graph on the HRV frequency parameters according to the optimal vs. non-optimal RSP: (**a**) VLF; (**b**) LF; (**c**) HF; (**d**) VLF power; (**e**) LF power; (**f**) HF power; (**g**) LF/HF ratio.

To confirm the pattern of all participants, we utilized the HRV frequency parameters, which are converted from 0 to 1 through normalization to remove individual differences. Figure 9 shows the difference between optimal and non-optimal respiration for HRV frequency parameters. Although VLF (%) did not show a clear difference compared to other parameters, the average value was slightly lower in the case of optimized breathing. On the other hand, VLF power showed a low value at the time of optimal respiration. LF (%) showed an overall low value at optimal respiration, and LF power also showed the same pattern. In addition, HF (%) clearly showed a pattern showing a high value at optimal respiration, but a clear result could not be confirmed in HF power. Finally, we confirmed an apparent pattern showing a low value for optimal respiration and a high value for non-optimal respiration in the LF/HF ratio.

## 5. Discussion

As mentioned in the review of the literature, inhalation inactivates the vagal tone, which immediately increases the heart rate. On the other hand, exhalation activates the vagal tone, which slows the heart rate due to the speed suppression of the sinus node [44]. In addition, some studies have shown that the rhythm synchronized with the heart was five to seven cycles per minute, within a frequency range of 0.01 to 0.15 Hz [45,46], and the rhythm synchronized with respiration was also similar to that of five to seven cycles [21,38,39]. The response of respiration and the heart can be affected by psychological or emotional factors.

Existing experimental results measuring the respiration and heart responses according to emotions from visual stimuli showed that the synchronization of respiration and heartbeat decreased in the arousal-negative state, which corresponds to the body’s response to stress [47]. Others suggested that there are differences in the rhythm of regular breathing between individuals [48,49,50].

From this point of view, this study proposed a process to find the breathing cycle optimized for the individual to improve the effectiveness of the RSA biofeedback, which consists of three processes: application, optimization, and validation. In the application process, we asked the subjects to breathe using five breathing cycles (9 s, 10 s, 11 s, 12 s, and 13 s) and acquired the PPG signals during this process. In the optimization process, we derived the HSI index after calculating the HF frequency through the collected data and selected the case with the highest HSI as the optimized RSP cycle per person. In the final validation step, we chose the seven stress-related indices as dependent variables by calculating the PPG signal, such as VLF (%), LF (%), HF (%), VLF power, LF power, HF power, and LF/HF ratio from the PPG signal. The experimental results show a statistical difference in the stress index in the optimal and non-optimal breathing case, and the tendency of the difference is consistent with the general tendency. Existing studies have shown that deep breathing has the effect of maintaining and changing a state of psychological comfort from negative states such as anxiety, stress, and anger, and its cycle affects the heart rhythm and activates the parasympathetic nervous system [51,52,53,54].

**Figure 10:**
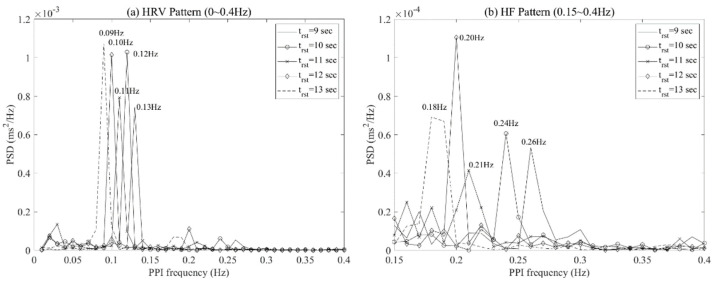
PPI frequency at maximum PSD for each respiratory cycle of a subject: HRV pattern (**a**) and HF pattern (**b**).

In the analysis on the frequency domain, harmonics means a multiple of the fundamental frequency. For example, if the fundamental frequency is 2 Hz, high harmonies occur at 4 Hz, 6 Hz, etc. Because HRV is periodic information of the RR interval through frequency analysis, it has harmonic property. According to previous studies, the spectral amplitude within the HF band (0.15–0.4 Hz) was used as an index of RSA [55,56,57]. When the power spectrum of the RR interval time series during controlled respiration was analyzed, a resonant peak was confirmed in the HF region [58,59]. In short, previous studies described that controlled respiration shows the dominant pattern not only in the LF region but also in the HF region. Our study confirmed the dominant pattern in the LF region, as shown in Figure 10a, which makes the HF region look like zero. However, we can see a dominant pattern when analyzing only the high-frequency region as Figure 10b. These results occurred at almost twice the fundamental frequency in the LF range.

**Figure 11:**
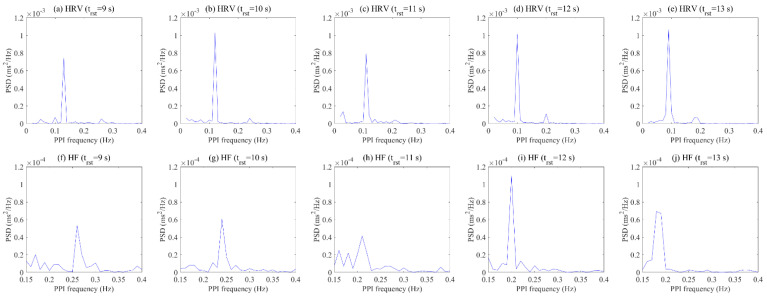
Comparison result of HRV region (**a**–**e**) and HF region (**f**–**j**) for each respiratory cycle of a subject.

The pattern of HRV is used to analyze frequency data transformed from PPI fluctuations and has components such as fundamental, second, third, etc., due to frequency harmony. When the subjects performed optimal respiration, existing studies selected the subject’s optimal respiration using a dominant pattern in the LF region of HRV, which is the fundamental component [21,60]. However, there are cases in which it is difficult to select the optimal respiration of a subject using only the maximum value for the fundamental component, as shown in Figure 11. Although the results show no significant difference when comparing the maximum values of HRV for each respiratory cycle, the second component of the HF range of HRV was able to confirm the dominant pattern in a specific respiratory cycle. These results reflect that the frequency of respiration is synchronized with the components in the HF region of HRV, as mentioned in the existing literature [55,56,57].

In this context, we assumed that HF power could be used to check whether the subject’s parasympathetic nervous system is currently activated according to respiratory cycles, and we observed the HF power of the subjects according to the breathing cycle. As a result, each subject showed a dominant pattern at a specific frequency in the HF power range according to the respiratory cycle. The pattern of HF power showed a difference not only between individuals, but also the breathing cycle within individuals. For this reason, we used HSI, a quantification index based on HF components, to determine the optimal respiration for each individual.

We tried to confirm the relationship between the HSI and the seven sympathetic and parasympathetic activation indices, which are linked to stress. Figure 6 shows a patterned visual change for other indicators, except for the power of VLF and VLF (%) according to the HSI. As the HSI increases, the power and percent of LF showed a decreasing pattern, while the power and percent of HF showed an increasing pattern. Finally, the LF/HF ratio showed a decreasing pattern as the HSI increased, which showed a relatively clearer linear pattern compared to other indicators. The results of linear regression analysis from a quantitative perspective indicate that r-squared was 0.55 for LF (%), 0.68 for LF power, 0.55 for HF (%), 0.52 for HF power, and 0.81 for LF/HF ratio. In other words, LF (%), LF power, and LF/HF ratio showed a negative linear relationship with the HSI, and HF (%) and HF power showed a positive linear relationship with it. As mentioned in Figure 3, the HSI is an index that quantifies the dominant pattern of peaks within the HF power range, which results from calculating the frequency change based on the PPI of the heart. A high value of HSI indicates the stable breathing and parasympathetic activation of the subjects. Therefore, we confirmed a linear pattern in the HRV frequency parameters as the HSI increased.

This study conducted a validation on the effect of the parasympathetic activation between the optimal RSP and non-optimal RSP for the individual based on the HSI. Table 1 shows statistical differences in all parameters, except VLF (%). In addition, Figure 8 shows that VLF power, LF (%), LF power, and LF/HF ratio show low values, while HF (%) and HF power show high values at optimal RSP. As mentioned in previous studies, these indicators are related to stress, and it was confirmed that they were effective in activating the parasympathetic nervous system when the subject breathed using the optimal RSP [61,62,63,64]. The visual patterns on all participants, as shown in Figure 9, showed a clear difference depending on the presence or absence of optimal respiration, except for VLF (%) and HF power.

## 6. Conclusions

This study proposed an overall process consisting of three steps—(1) application, (2) optimization, and (3) validation—to find an optimized respiratory cycle. We defined a performance index, or heart stabilization indicator (HSI), using data in the high-frequency (HF) range of heart rate variability (HRV) for selecting an optimized breathing cycle.

In the first step, we measured the PPG data of the subjects based on the respiratory guidelines with random breathing cycles. Then, after calculating the HSI index using the proposed formulation and the PPG data, we selected the respiratory cycle showing the highest HSI index as the optimal respiratory cycle. In the last step, we verified the proposed method by comparatively analyzing the stress index related to the HRV frequency parameters in the case of optimal and non-optimal respiratory cycles.

The experimental result in the two cases shows that there is a similar pattern compared with the previous studies from the perspective of the parasympathetic activation. Furthermore, there is a clear and significant statistical difference for the stress indices, except for VLF. In other words, according to the increase in the HSI, we confirmed that LF (%), LF power, and LF/HF ratio decreased, while HF (%) and HF power increased.

Our findings clearly show that the HSI index can be a significant index for finding optimal respiration. The optimal respiration derived through the index can be applied as a reference value for the feedback, which is one of the main factors in biofeedback. Since the HSI indicator in this study is generated through resonance respiration, it is expected that there is the possibility for future research using it as a factor to check heart conditions such as arrhythmias as well as in the field of RSA biofeedback. Finally, we expect that the proposed design in this paper will used as an alternative for improving the effectiveness of RSA biofeedback.

## Figures and Tables

**Table 1:** Wilcoxon signed-rank test between optimal and non-optimal RSP.

Variable	Optimal RSP	Non-Optimal RSP	Z	*p*
	M (SD)	M (SD)		
VLF (%)	8.1359 (7.6382)	8.5199 (5.9367)	−1.062	0.288
LF (%)	27.7045 (19.4434)	44.6741 (16.2995)	−6.505	0.000
HF (%)	64.1596 (22.2799)	46.806 (17.6863)	−6.316	0.000
VLF Power	0.0004 (0.0004)	0.0005 (0.0003)	−3.246	0.001
LF Power	0.0016 (0.0018)	0.0029 (0.0018)	−6.203	0.000
HF Power	0.0043 (0.0038)	0.0035 (0.0029)	−2.644	0.008
LF/HF ratio	1.1185 (3.021)	3.2043 (3.9283)	−6.791	0.000

## Data Availability

Data available on request due to restrictions.
